# Prospective study of colorectal cancer risk and physical activity, diabetes, blood glucose and BMI: exploring the hyperinsulinaemia hypothesis

**DOI:** 10.1054/bjoc.2000.1582

**Published:** 2001-02

**Authors:** T I Lund Nilsen, L J Vatten

**Affiliations:** Department of Community Medicine and General Practice, Norwegian University of Science and Technology, University Medical Center, Trondheim, N-7489, Norway; Norwegian Cancer Society, PO Box 5327 Majorstua, Oslo, N-0304, Norway

**Keywords:** colorectal cancer, lifestyle factors, hyperinsulinaemia, risk

## Abstract

A sedentary lifestyle, obesity, and a Westernized diet have been implicated in the aetiology of both colorectal cancer and non-insulin dependent diabetes mellitus, leading to the hypothesis that hyperinsulinaemia may promote colorectal cancer. We prospectively examined the association between colorectal cancer risk and factors related to insulin resistance and hyperinsulinaemia, including BMI, physical activity, diabetes mellitus, and blood glucose, in a cohort of 75 219 Norwegian men and women. Information on incident cases of colorectal cancer was made available from the Norwegian Cancer Registry. Reported *P* values are two-sided. During 12 years of follow up, 730 cases of colorectal cancer were registered. In men, but not in women, we found a negative association with leisure-time physical activity (*P* for trend = 0.002), with an age-adjusted RR for the highest versus the lowest category of activity of 0.54 (95% CI = 0.37–0.79). Women, but not men, with a history of diabetes were at increased risk of colorectal cancer (age-adjusted RR = 1.55; 95% CI = 1.04–2.31), as were women with non-fasting blood glucose ≥8.0 mmol l^−1^(age-adjusted RR = 1.98; 95% CI = 1.31–2.98) compared with glucose <8.0 mmol l^−1^. Overall, we found no association between BMI and risk of colorectal cancer. Additional adjustment including each of the main variables, marital status, and educational attainment did not materially change the results. We conclude that the inverse association between leisure-time physical activity and colorectal cancer in men, and the positive association between diabetes, blood glucose, and colorectal cancer in women, at least in part, support the hypothesis that insulin may act as a tumour promoter in colorectal carcinogenesis. © 2001 Cancer Research Campaign http://www.bjcancer.com


British Journal of Cancer (2001) **84**(3), 417–422
© 2001 Cancer Research Campaign
doi: 10.1054/ bjoc.2000.1582, available online at http://www.idealibrary.com on IDEAL®

http://www.bjcancer.com

### Prospective study of colorectal cancer risk and physical activity, diabetes, blood glucose and BMI: exploring the hyperinsulinaemia hypothesis

**TI Lund Nilsen[1,2] and LJ Vatten[1]**

[1]Department of Community Medicine and General Practice, Norwegian University of Science and Technology, University Medical Center, N-7489 Trondheim, Norway; [2]Norwegian Cancer Society, PO Box 5327 Majorstua, N-0304 Oslo, Norway

**Summary** A sedentary lifestyle, obesity, and a Westernized diet have been implicated in the aetiology of both colorectal cancer and non-insulin dependent diabetes mellitus, leading to the hypothesis that hyperinsulinaemia may promote colorectal cancer. We prospectively examined the association between colorectal cancer risk and factors related to insulin resistance and hyperinsulinaemia, including BMI, physical activity, diabetes mellitus, and blood glucose, in a cohort of 75 219 Norwegian men and women. Information on incident cases of colorectal cancer was made available from the Norwegian Cancer Registry. Reported *P* values are two-sided. During 12 years of follow up, 730 cases of colorectal cancer were registered. In men, but not in women, we found a negative association with leisure-time physical activity (*P* for trend = 0.002), with an age-adjusted RR for the highest versus the lowest category of activity of 0.54 (95% CI = 0.37–0.79). Women, but not men, with a history of diabetes were at increased risk of colorectal cancer (age-adjusted RR = 1.55; 95% CI = 1.04–2.31), as were women with non-fasting blood glucose ≥8.0 mmol $l^{-1}$ (age-adjusted RR = 1.98; 95% CI = 1.31–2.98) compared with glucose <8.0 mmol $l^{-1}$. Overall, we found no association between BMI and risk of colorectal cancer. Additional adjustment including each of the main variables, marital status, and educational attainment did not materially change the results. We conclude that the inverse association between leisure-time physical activity and colorectal cancer in men, and the positive association between diabetes, blood glucose, and colorectal cancer in women, at least in part, support the hypothesis that insulin may act as a tumour promoter in colorectal carcinogenesis. © 2001 Cancer Research Campaign http://www.bjcancer.com

**Keywords**: colorectal cancer; lifestyle factors; hyperinsulinaemia; risk

Obesity (Giovannucci et al, 1995, 1996; Martinez et al, 1997), physical inactivity (Colditz et al, 1997), and consumption of a Western diet high in fat and low in fibre (Willett, 1989) are associated with an increased risk of colorectal cancer. These lifestyle factors have also been associated with an increased risk of non-insulin-dependent diabetes mellitus (Colditz et al, 1990; Manson et al, 1991, 1992; Salmeron et al, 1997a, b). Based on the similarity of risk factors for colorectal cancer and diabetes mellitus, McKeown-Eyssen (1994) and Giovannucci (1995) have suggested the two associations to be biologically related. They propose that dietary and exercise factors may lead to insulin resistance and hyperinsulinaemia, and that the increased level of insulin may stimulate the growth of colorectal tumours.

On this background, we prospectively examined the association between obesity, physical activity, diabetes mellitus, and blood glucose and the risk of colorectal cancer in a cohort of 75 219 Norwegian men and women.

### MATERIALS AND METHODS

### The cohort

The study cohort was established between 1984 and 1986, when the National Health Screening Service in Norway conducted the Nord-Trøndelag Health Survey. All residents in Nord-Trøndelag county aged 20 years or older were invited to participate in the survey, which included a health examination with different anthropometric and physiological measures, and two questionnaires providing information on medical history and lifestyle factors. Among 85 100 eligible persons, 77 310 (90.8%) filled in the questionnaire that was mailed with the invitation. A more comprehensive description of the participants, questionnaires, and screening procedures is previously given by Holmen and Midthjell (1990).

### Follow-up

The unique 11-digit identity number of every citizen in Norway enabled linkage between the Nord-Trøndelag database and the Norwegian Cancer Registry in order to identify incident cases of colorectal cancer in the cohort. To improve ascertainment and quality, the Cancer Registry data are matched against the Register of Deaths at Statistics Norway. For all cancer cases registered since 1953, 84.7% were histologically verified and only 1.7% of the diagnoses have been based on death certificate alone (The Cancer Registry of Norway, 1998). The participants in the present study included 38 244 women and 36 975 men aged 20 years and more at baseline, who had no history of any cancer at study entry. Each participant contributed person-years from the date of study entry (January 1984–April 1986) until the date of cancer diagnosis (at any site), death, emigration, or the cut-off date of 1 January 1996, whichever occurred first.

*Received 12 July 2000*
*Revised 16 October 2000*
*Accepted 16 October 2000*

*Correspondence to:* TI Lund Nilsen

### Study factors

Standardized measurements of body height and weight obtained at the health examination were used to calculate body mass index (BMI) as weight in kilograms divided by the squared value of height in metres (kg/m$^2$), and subsequently categorized into quartiles.

In the health survey questionnaire on leisure-time physical activity, the participants were asked, 'how often do you exercise?' 'how hard do you exercise?', and 'for how long do you carry on?', with five, three, and four response choices, respectively. With respect to frequency, we considered exercising less than once a week as low frequency, whereas exercising 1–3 times per week and more than 3 times per week were classified as medium and high frequency. In addition, we used the information on frequency, intensity, and duration to calculate a summary index of physical activity, and categorized this into tertiles of low, medium, and high activity. History of diabetes mellitus (both insulin-dependent and non-insulin-dependent) was assessed from the baseline questionnaire, whereas non-fasting blood glucose was dichotomized at ≥8.0 mmol l$^{-1}$ according to the cut point for a 'positive screening' used in the health survey (Holmen and Midthjell, 1990), which is in conformity with the WHO criteria of 1980 (WHO, 1980).

### Statistical analysis

Analyses were conducted separately for males and females. The Cox proportional hazards model (Kleinbaum, 1995) was used to examine the association between colorectal cancer and each of the variables under study. Age within 10 categories (<40, 40–44, ..., 75–79, and ≥80) and the relevant exposure variable were included as independent variables, and individual number of person-years as the dependent variable. This produced age-adjusted incidence rate ratios as estimates of the relative risk (RR) with 95% confidence intervals (CIs). When appropriate, a two-sided test for trend across exposure categories was calculated by treating the categories as ordinal variables in the proportional hazards model. We analysed colon cancer and rectal cancer separately, as well as associations with colon cancer that presented with metastases.

Multivariate analyses were conducted to assess potential confounding with other factors. In addition to each main variable (BMI, physical activity, diabetes, and blood glucose), confounding with marital status and educational attainment was considered. Due to potential co-linearity, diabetes and blood glucose were not entered simultaneously into the multivariate analyses.

All statistical analyses were performed using the statistical software SPSS for Windows (Release 8.0.0, Copyright © SPSS Inc, 1989–1997).

### RESULTS

During 12 years of follow-up (median = 10.8), a total of 730 cases of colorectal cancer developed in 751 922 person-years. Among males, 234 colon cancers and 128 rectal cancers were diagnosed, whereas 277 colon cancers and 91 rectal cancers developed in females (Table 1). Mean age at study entry was 48.5 years (range = 20–100 years) among men and 49.8 years (range = 20–101 years) among women, while mean age at diagnosis of colorectal cancer was 71.5 years (range = 36–93 years) among men and 71.9 years (range = 35–97 years) among women.

Overall, we observed no significant association between BMI and risk of colorectal cancer (Table 2). Frequency of leisure-time physical activity was inversely associated with the risk of colorectal cancer in men (*P* for trend = 0.04), but not in women (*P* for trend = 0.85). Compared with the least active men, those with the highest index of physical activity had a nearly 50% lower risk of colorectal cancer (RR = 0.54; 95% CI = 0.37–0.79), and this negative association was slightly stronger for colon cancer (RR = 0.47; 95% CI = 0.28–0.80) than for rectal cancer (RR = 0.63; 95% CI = 0.36–1.12) (data not shown).

No significant association with diabetes mellitus was found among men (RR = 0.66; 95% CI = 0.35–1.24), but women who reported diabetes mellitus at baseline had 55% higher risk of colorectal cancer than women without diabetes (RR = 1.55; 95% CI = 1.04–2.31). Similarly, women with non-fasting blood glucose equal to or above 8.0 mmol l$^{-1}$ had twice the risk of women with lower values (RR = 1.98; 95% CI = 1.31–2.98). Roughly, the positive association with diabetes in women was similar for colon (RR = 1.60; 95% CI = 1.02–2.51) and rectal cancer (1.41; 95% CI = 0.61–3.27), while the association with blood glucose was stronger for rectal (RR = 2.70; 95% CI = 1.29–5.61) than for colon cancer (RR = 1.76; 95% CI = 1.07–2.88) (data not shown).

We also conducted secondary analyses restricted to 346 colon cancer cases that presented with metastatic disease at diagnosis (162 males and 184 females) (Table 3). Among men, the negative association with physical activity was strengthened; the most active had nearly 70% lower risk than the least active (RR = 0.33; 95% CI = 0.16–0.67). The increased risk in women associated with diabetes mellitus was not present for metastatic disease (RR = 1.12; 95% CI = 0.59–2.14), but the positive association with non-fasting blood glucose persisted (RR = 1.92; 95% CI = 1.06–3.47).

For each study variable, we compared age-adjusted and multivariate adjusted associations to assess potential confounding with any of the other main variables, and with marital status and educational attainment, but the results were not materially different (data not shown).

### DISCUSSION

Our main findings – that risk of colorectal cancer was inversely associated with physical activity in men, and positively associated with diabetes mellitus and high blood glucose in women – support the hypothesis that hyperinsulinaemia may stimulate the growth of colorectal tumours. However, the gender specific discrepancy in the results cannot be readily explained, and calls for cautious interpretation.

Several cohort studies have shown that physical activity is negatively associated with colorectal cancer risk, and in accordance with our findings in men, a reduction of 40 to 50% has been reported (Wu et al, 1987; Lee et al, 1991; Giovannucci et al, 1995, 1996; Thune and Lund, 1996; Martinez et al, 1997). Two interpretations have been proposed for the reduced risk. First, physical activity stimulates colon peristalsis and decreases bowel transit time (Cordain et al, 1986), and this may reduce exposure to carcinogens. Second, physical activity may increase insulin sensitivity (Koivisto et al, 1986) and reduce plasma insulin (Regensteiner et al, 1991). Insulin is a colon tumour promoter in rats (Tran et al, 1996), and in vitro, insulin is a mitogen for colon carcinoma cells (Koenuma et al, 1989).

Contrary to some other studies, we found no significant association with physical activity among women (Thune and Lund, 1996; Martinez et al, 1997). We also explored the effect of extreme

© 2001 Cancer Research Campaign

**Table 1** Follow-up[a] of 75 219 Norwegian men and women participating in the Nord-Trøndelag health survey 1984–1986

| | Men | | | | Women | | | |
|---|---|---|---|---|---|---|---|---|
| Age[b] (years) | Subjects[c] | Person-years[d] | Colon[e] | Rectal[f] | Subjects[c] | Person-years[d] | Colon[e] | Rectal[f] |
| <50 | 20 213 | 218 420 | 22 | 7 | 20 043 | 216 936 | 31 | 10 |
| 50–59 | 5540 | 56 882 | 38 | 26 | 5465 | 57 319 | 40 | 13 |
| 60–69 | 6034 | 55 086 | 81 | 45 | 6080 | 60 759 | 81 | 35 |
| 70–79 | 3818 | 28 328 | 69 | 39 | 4556 | 39 117 | 89 | 24 |
| ≥80 | 1370 | 6992 | 24 | 11 | 2100 | 12 083 | 36 | 9 |
| Total | 36 975 | 365 708 | 234 | 128 | 38 244 | 386 214 | 277 | 91 |

[a]Censored by cancer diagnosis (all sites), death, emigration, or cut-off 1 January 1996. [b]Age at entry to the study. [c]Number of individuals included at baseline. [d]Accumulated person-years during 12 years of follow-up. [e]Number of incident cases of colon cancer reported to the Cancer Registry of Norway. [f]Number of incident cases of rectal cancer reported to the Cancer Registry of Norway.

**Table 2** Age-adjusted relative risks (RRs) with 95% confidence intervals (CIs) and *P* values for trend[a] of incident colorectal cancer among 75 219 Norwegian men and women participating in the Nord-Trøndelag health survey, 1984–1986: associations with BMI, physical activity, diabetes mellitus, and blood glucose

| | | Men | | | | Women | | | |
|---|---|---|---|---|---|---|---|---|---|
| Variables[b] | | Cases | Person-years | RR | 95% CI | Cases | Person-years | RR | 95% CI |
| BMI | | | | | | | | | |
| men | women | | | | | | | | |
| ≤23.0 | ≤21.8 | 75 | 89 694 | 1.0 | Reference | 57 | 96 249 | 1.0 | Reference |
| 23.1–24.9 | 21.9–24.2 | 71 | 90 360 | 0.90 | 0.65–1.24 | 69 | 95 472 | 0.89 | 0.63–1.27 |
| 25.0–27.1 | 24.3–27.4 | 91 | 88 804 | 0.96 | 0.71–1.30 | 90 | 92 789 | 0.80 | 0.57–1.13 |
| ≥27.2 | ≥27.5 | 117 | 86 489 | 1.07 | 0.80–1.42 | 142 | 90 400 | 0.98 | 0.71–1.34 |
| | | | | *P* = 0.51 | | | | *P* = 0.96 | |
| Frequency of physical activity | | | | | | | | | |
| Low | | 118 | 121 217 | 1.0 | Reference | 128 | 122 579 | 1.0 | Reference |
| Medium | | 130 | 134 990 | 0.99 | 0.77–1.27 | 103 | 149 925 | 0.81 | 0.62–1.05 |
| High | | 55 | 37 140 | 0.69 | 0.50–0.95 | 62 | 38 650 | 1.12 | 0.83–1.52 |
| | | | | *P* = 0.04 | | | | *P* = 0.85 | |
| Physical activity index | | | | | | | | | |
| Low | | 100 | 61 456 | 1.0 | Reference | 92 | 75 910 | 1.0 | Reference |
| Medium | | 78 | 67 534 | 0.87 | 0.65–1.17 | 55 | 66 410 | 0.95 | 0.68–1.33 |
| High | | 35 | 66 864 | 0.54 | 0.37–0.79 | 32 | 64 268 | 0.81 | 0.54–1.23 |
| | | | | *P* = 0.002 | | | | *P* = 0.34 | |
| History of diabetes mellitus | | | | | | | | | |
| No | | 349 | 357 986 | 1.0 | Reference | 340 | 376 715 | 1.0 | Reference |
| Yes | | 10 | 6510 | 0.66 | 0.35–1.24 | 27 | 8202 | 1.55 | 1.04–2.31 |
| Blood glucose levels (mmol $l^{-1}$) | | | | | | | | | |
| <8.0 | | 321 | 182 295 | 1.0 | Reference | 316 | 208 261 | 1.0 | Reference |
| ≥8.0 | | 21 | 11 302 | 0.90 | 0.58–1.40 | 25 | 6502 | 1.98 | 1.31–2.98 |

[a]Two-sided *P* values for trend by proportional hazards model when variables were treated as ordinal variables. [b]Information on each variable was not available on all participants.

© 2001 Cancer Research Campaign

**Table 3** Age-adjusted relative risks (RRs) with 95% confidence intervals (CIs) and *P* values for trend[a] of metastatic colon cancer among 75 219 Norwegian men and women participating in the Nord-Trøndelag health survey, 1984–1986: associations with BMI, physical activity, diabetes mellitus, and blood glucose

| | | Men | | | | Women | | | |
|---|---|---|---|---|---|---|---|---|---|
| Variables[b] | | Cases | Person-years | RR | 95% CI | Cases | Person-years | RR | 95% CI |
| BMI | | | | | | | | | |
| men | women | | | | | | | | |
| ≤23.0 | ≤21.8 | 36 | 89 694 | 1.0 | Reference | 27 | 96 249 | 1.0 | Reference |
| 23.1–24.9 | 21.9–24.2 | 23 | 90 360 | 0.61 | 0.36–1.02 | 33 | 95 472 | 0.91 | 0.55–1.51 |
| 25.0–27.1 | 24.3–27.4 | 42 | 88 804 | 0.93 | 0.59–1.45 | 44 | 92 789 | 0.84 | 0.52–1.37 |
| ≥27.2 | ≤27.5 | 58 | 86 489 | 1.11 | 0.73–1.68 | 75 | 90 400 | 1.11 | 0.71–1.75 |
| | | | | | $P = 0.25$ | | | | $P = 0.48$ |
| Frequency of physical activity | | | | | | | | | |
| Low | | 56 | 121 217 | 1.0 | Reference | 68 | 122 579 | 1.0 | Reference |
| Medium | | 51 | 134 990 | 0.82 | 0.56–1.20 | 48 | 149 925 | 0.71 | 0.49–1.04 |
| High | | 29 | 37 140 | 0.76 | 0.48–1.20 | 28 | 38 650 | 0.95 | 0.61–1.47 |
| | | | | | $P = 0.20$ | | | | $P = 0.47$ |
| Physical activity index | | | | | | | | | |
| Low | | 43 | 61 456 | 1.0 | Reference | 47 | 75 910 | 1.0 | Reference |
| Medium | | 37 | 67 534 | 0.96 | 0.62–1.49 | 22 | 66 410 | 0.73 | 0.44–1.22 |
| High | | 9 | 66 864 | 0.33 | 0.16–0.67 | 16 | 64 268 | 0.77 | 0.43–1.38 |
| | | | | | $P = 0.005$ | | | | $P = 0.27$ |
| History of diabetes mellitus | | | | | | | | | |
| No | | 155 | 357 986 | 1.0 | Reference | 174 | 376 715 | 1.0 | Reference |
| Yes | | 6 | 6510 | 0.88 | 0.39–1.99 | 10 | 8202 | 1.12 | 0.59–2.14 |
| Blood glucose levels (mmol $l^{-1}$) | | | | | | | | | |
| <8.0 | | 146 | 182 295 | 1.0 | Reference | 155 | 208 261 | 1.0 | Reference |
| ≥8.0 | | 7 | 11 302 | 0.66 | 0.31–1.40 | 12 | 6502 | 1.92 | 1.06–3.47 |

[a]Two-sided *P* values for trend by proportional hazards model when variables were treated as ordinal variables. [b]Information on each variable was not available on all participants.

*© 2001 Cancer Research Campaign*

levels of physical activity in women, but this did not change the results. Misclassification of physical activity due to energy expenditure in housework rather than in leisure-time activity might contribute to the null finding in women, and little variation in physical activity could mask a difference in risk. Also, physically active women could be more health conscious and more likely to seek medical advice for early symptoms, which may lead to higher detection of early-stage cancer. Furthermore, physically active individuals may eat less saturated fat and more fibre than less active people. However, an inverse association with colorectal cancer risk has been shown, also after adjustment for dietary intake of saturated fat, red meat and fibre (Whittemore et al, 1990; Giovannucci et al, 1995).

Previous studies have shown no consistent association between diabetes mellitus and risk of colorectal cancer (Ragozzino et al, 1982; O'Mara et al, 1985; La  et al, 1991; LaVecchia et al, 1997; Le Marchand et al, 1997), but recently, two prospective studies reported a positive association (Will et al, 1998; Hu et al, 1999). In our study, there was also a positive association with diabetes, but only among women. Several possibly causative mechanisms have been suggested; diabetes may slow down bowel transit (Iber et al, 1993); production of bile acids that promote colon carcinogenesis may increase (Narisawa et al, 1974; Nakamura et al, 1993); and high insulin levels may promote colon tumour growth (McKeown-Eyssen, 1994; Giovannucci, 1995). Nonetheless, these factors cannot explain that a positive association with diabetes is present only among women. In this study, few men with diabetes developed colorectal cancer, and the statistical power to examine this question may be to low. Further, the positive association with diabetes in women may be a result of increased medical surveillance.

McKeown-Eyssen (1994) has suggested serum triglycerides and plasma glucose to be involved in colorectal carcinogenesis, possibly by increasing insulin secretion. In our study, we found a two-fold increased risk among women with a non-fasting blood glucose of 8.0 mmol $l^{-1}$ or higher, which is in agreement with a recent study by Schoen et al (1999). In contrast to our results, they also found a similar association in men.

Obesity may be a determinant of insulin resistance and hyperinsulinaemia (Bjorntorp, 1991). Some studies support a role of obesity in male colorectal cancer (Whittemore et al, 1990; Giovannucci et al, 1995, 1996; LeMarchand et al, 1997; Martinez et al, 1997), and a weak positive association has been found in women (Phillips and Snowdon, 1985; Wu et al, 1987). Our results showed, however, no association with BMI in either gender. A prediagnostic weight loss could disturb an association between obesity and colorectal cancer, but excluding persons diagnosed with colorectal cancer within the first three years of follow-up did not materially change the results.

In summary, these results may, at least in part, support the hyperinsulinaemia hypothesis in colorectal cancer (McKeown-Eyssen, 1994; Giovannucci, 1995). We found a clear risk reduction associated with high leisure-time physical activity in men, and among women, we found that diabetes and high blood glucose were associated with increased risk.

### ACKNOWLEDGEMENTS

This research is based on data made available by the National Health Screening Service, The Cancer Registry of Norway, and the National Institute of Public Health, Community Medicine Research Centre in Verdal, Nord-Trøndelag County, Norway.

TI Lund Nilsen is a recipient of a research fellowship from the Norwegian Cancer Society, Oslo, Norway.

### REFERENCES

Bjorntorp P (1991) Metabolic implications of body fat distribution. *Diabetes Care* **14**: 1132–1143

Colditz GA, Willett WC, Stampfer MJ, Manson JE, Hennekens CH, Arky RA and Speizer FE (1990) Weight as a risk factor for clinical diabetes in women. *Am J Epidemiol* **132**: 501–513

Colditz GA, Cannuscio CC and Frazier AL (1997) Physical activity and reduced risk of colon cancer: implications for prevention. *Cancer Causes Control* **8**: 649–667

Cordain L, Latin RW and Behnke JJ (1986) The effects of an aerobic running program on bowel transit time. *J Sports Med Phys Fitness* **26**: 101–104

Giovannucci E (1995) Insulin and colon cancer. *Cancer Causes Control* **6**: 164–179

Giovannucci E, Ascherio A, Rimm EB, Colditz GA, Stampfer MJ and Willett WC (1995) Physical activity, obesity, and risk for colon cancer and adenoma in men. *Ann Intern Med* **122**: 327–334

Giovannucci E, Colditz GA, Stampfer MJ and Willett WC (1996) Physical activity, obesity, and risk of colorectal adenoma in women (United States). *Cancer Causes Control* **7**: 253–263

Holmen J and Midthjell K (1990) *The Nord-Trøndelag health survey 1984–86: purpose, background and methods: participation, non-participation and frequency*. Report no 4, National Institute of Public Health: Oslo

Hu FB, Manson JE, Liu S, Hunter D, Colditz GA, Michels KB, Speizer FE and Giovannucci E (1999) Prospective study of adult onset diabetes mellitus (type 2) and risk of colorectal cancer in women. *J Natl Cancer Inst* **91**: 542–547

Iber FL, Parveen S, Vandrunen M, Sood KB, Reza F, Serlovsky R and Reddy S (1993) Relation of symptoms to impaired stomach, small bowel, and colon motility in long-standing diabetes. *Dig Dis Sci* **38**: 45–50

Kleinbaum DG (1995) *Survival analysis: a self-learning text*. Springer-Verlag: New York

Koenuma M, Yamori T and Tsuruo T (1989) Insulin and insulin-like growth factor 1 stimulate proliferation of metastatic variants of colon carcinoma 26. *Jpn J Cancer Res* **80**: 51–58

Koivisto VA, Yki-Jarvinen H and DeFronzo RA (1986) Physical training and insulin sensitivity. *Diabetes Metab Rev* **1**: 445–481

La Vecchia C, D'Avanzo B, Negri E and Franceschi S (1991) History of selected diseases and the risk of colorectal cancer. *Eur J Cancer* **27**: 582–586

La Vecchia C, Negri E, Decarli A and Franceschi S (1997) Diabetes mellitus and colorectal cancer risk. *Cancer Epidemiol Biomarkers Prev* **6**: 1007–1010

Le Marchand L, Wilkens LR, Kolonel LN, Hankin JH and Lyu LC (1997) Associations of sedentary lifestyle, obesity, smoking, alcohol use, and diabetes with the risk of colorectal cancer. *Cancer Res* **57**: 4787–4794

Lee IM, Paffenbarger RS Jr and Hsieh C (1991) Physical activity and risk of developing colorectal cancer among college alumni. *J Natl Cancer Inst* **83**: 1324–1329

Manson JE, Rimm EB, Stampfer MJ, Colditz GA, Willett WC, Krolewski AS, Rosner B, Hennekens CH and Speizer FE (1991) Physical activity and incidence of non-insulin-dependent diabetes mellitus in women. *Lancet* **338**: 774–778

Manson JE, Nathan DM, Krolewski AS, Stampfer MJ, Willett WC and Hennekens CH (1992) A prospective study of exercise and incidence of diabetes among US male physicians. *JAMA* **268**: 63–67

Martinez ME, Giovannucci E, Spiegelman D, Hunter DJ, Willett WC and Colditz GA (1997) Leisure-time physical activity, body size, and colon cancer in women. Nurses' Health Study Research Group. *J Natl Cancer Inst* **89**: 948–955

McKeown-Eyssen G (1994) Epidemiology of colorectal cancer revisited: are serum triglycerides and/or plasma glucose associated with risk? *Cancer Epidemiol Biomarkers Prev* **3**: 687–695

Nakamura T, Imamura K, Kasai F, Tsushima F, Kikuchi H and Takebe K (1993) Fecal excretions of hydroxy fatty acid and bile acid in diabetic diarrheal patients. *J Diabetes Complications* **7**: 8–11

Narisawa T, Magadia NE, Weisburger JH and Wynder EL (1974) Promoting effect of bile acids on colon carcinogenesis after intrarectal instillation of N-methyl-N′-nitro-N-nitrosoguanidine in rats. *J Natl Cancer Inst* **53**: 1093–1097

O'Mara BA, Byers T and Schoenfeld E (1985) Diabetes mellitus and cancer risk: a multisite case-control study. *J Chronic Dis* **38**: 435–441

© 2001 Cancer Research Campaign

Phillips RL and Snowdon DA (1985) Dietary relationships with fatal colorectal cancer among Seventh-Day Adventists. *J Natl Cancer Inst* **74**: 307–317

Ragozzino M, Melton LJ 3rd, Chu CP and Palumbo PJ (1982) Subsequent cancer risk in the incidence cohort of Rochester, Minnesota, residents with diabetes mellitus. *J Chronic Dis* **35**: 13–19

Regensteiner JG, Mayer EJ, Shetterly SM, Eckel RH, Haskell WL, Marshall JA, Baxter J and Hamman RF (1991) Relationship between habitual physical activity and insulin levels among nondiabetic men and women. San Luis Valley Diabetes Study. *Diabetes Care* **14**: 1066–1074

Salmeron J, Ascherio A, Rimm EB, Colditz GA, Spiegelman D, Jenkins DJ, Stampfer MJ, Wing AL and Willett WC (1997a) Dietary fiber, glycemic load, and risk of NIDDM in men. *Diabetes Care* **20**: 545–550

Salmeron J, Manson JE, Stampfer MJ, Colditz GA, Wing AL and Willett WC (1997b). Dietary fiber, glycemic load, and risk of non-insulin-dependent diabetes mellitus in women. *JAMA* **277**: 472–477

Schoen RE, Tangen CM, Kuller LH, Burke GL, Cushman M, Tracy RP, Dobs A and Savage PJ (1999) Increased blood glucose and insulin, body size, and incident colorectal cancer. *J Natl Cancer Inst* **91**: 1147–1154

The Cancer Registry of Norway (1998) *Cancer in Norway 1995*. The Cancer Registry of Norway: Oslo

Thune I and Lund E (1996) Physical activity and risk of colorectal cancer in men and women. *Br J Cancer* **73**: 1134–1140

Tran TT Medline A and Bruce WR (1996) Insulin promotion of colon tumors in rats. *Cancer Epidemiol Biomarkers Prev* **5**: 1013–1015

Whittemore AS, Wu-Williams AH, Lee M, Zheng S, Gallagher RP, Jiao DA, Zhou L, Wang XH, Chen K and Jung D, et al (1990) Diet, physical activity, and colorectal cancer among Chinese in North America and China. *J Natl Cancer Inst* **82**: 915–926

WHO (1980) *WHO expert committee on diabetes mellitus, Second report*. Technical report series no 646, World Health Organization: Geneva

Will JC, Galuska DA, Vinicor F and Calle EE (1998) Colorectal cancer: another complication of diabetes mellitus? *Am J Epidemiol* **147**: 816–825

Willett W (1989) The search for the causes of breast and colon cancer. *Nature* **338**: 389–394

Wu AH, Paganini-Hill A, Ross RK and Henderson BE (1987) Alcohol, physical activity and other risk factors for colorectal cancer: a prospective study. *Br J Cancer* **55**: 687–694

*© 2001 Cancer Research Campaign*
